# Comparing phenotypic features between Parkinson’s disease dementia and dementia with Lewy bodies

**DOI:** 10.1007/s00415-026-13649-9

**Published:** 2026-02-10

**Authors:** Chaminda Withanachchi Gunawardana, Peter G. R. Burke, Anna Ignatavicius, Elie Matar, Simon J. G. Lewis

**Affiliations:** 1 Faculty of Medicine, Health and Human Sciences, Parkinson’s Disease Research Clinic, Macquarie University, Sydney, NSW 2109 Australia; 2 Faculty of Medicine and Health, Sydney Medical School, University of Sydney, Camperdown, NSW 2050 Australia; 3 Department of Neurology, Royal Prince Alfred Hospital, Camperdown, NSW 2050 Australia; 4 Faculty of Medicine, Health and Human Sciences, Macquarie Medical School, Macquarie University, Sydney, NSW 2109 Australia

**Keywords:** Parkinson’s disease dementia, Dementia with Lewy bodies, Psychosis, Hallucinations, Delusions, Neuropsychiatric symptoms

## Abstract

**Background:**

Parkinson's disease dementia (PDD) and dementia with Lewy bodies (DLB) are two synucleinopathies that share considerable overlap in their clinical and pathological characteristics.

**Objective:**

This study aimed to evaluate the profile of phenotypic features across PDD and DLB to highlight their shared and distinctive features to explore any possible neurobiological underpinnings.

**Methods:**

Patients diagnosed with Parkinson's disease dementia (PDD) (n=31) and dementia with Lewy bodies (DLB) (n=30) underwent evaluation for motor and non-motor symptoms using the International Parkinson and Movement Disorder Society -Unified Parkinson's Disease Rating Scale, the Scales for Outcomes in Parkinson’s Disease-Psychiatric Complications, the Montreal Cognitive Assessment, the Hospital Anxiety and Depression Scale and the Epworth Sleepiness Scale.

**Results:**

Patients with PDD showed a more severe phenotype than those with DLB, with higher scores in motor severity, hallucinations, delusions, somnolence, and urinary dysfunction (all p≤0.01). The correlations between neuropsychiatric symptoms differed between groups. In both PDD and DLB, depression correlated with anxiety (PDD ρ=0.54; DLB ρ=0.65; both p<0.001). In PDD, depression was additionally correlated with delusions (ρ=0.47; p<0.001), apathy (ρ=0.41; p<0.001), and sexual preoccupation (ρ=0.35; p<0.001), whereas in DLB depression correlated with hallucinations (ρ=0.38; p<0.001). PDD patients showed greater impairment in activities of daily living (ADLs) than DLB patients (p<0.001), correlating with motor severity (ρ=0.65; p<0.001) and fatigue (ρ=0.58; p<0.001). In DLB, impaired ADLs were linked to hallucinations (ρ=0.39; p<0.05), poorer cognition (ρ=0.47; p<0.01), urinary dysfunction (ρ=0.38; p<0.001), and fatigue (ρ=0.45; p<0.001).

**Conclusion:**

Despite being grouped under Lewy body dementias, these findings show PDD and DLB have distinct phenotypes reflecting their neurobiological underpinnings. This has implications for symptomatic and disease-modifying management. Future research should explore pathological mechanisms of these characteristics to improve diagnosis and treatment strategies.

## Introduction

Parkinson’s disease dementia (PDD) and dementia with Lewy bodies (DLB) are collectively referred to as the Lewy body dementias (LBDs) and constitute the second most prevalent cause of dementia globally [1, 2]. Both diseases are synucleinopathies and are characterised by the pathological accumulation of the protein α-synuclein [3]. Owing to the considerable overlap in clinical and pathological characteristics, there is an ongoing debate regarding whether PDD and DLB represent a single entity or two distinct conditions [4, 5] Currently, the diagnostic criteria distinguishing between these LBDs has relied on the highly contentious and arbitrary, one-year cutoff rule [6, 7]. Specifically, if the dementia manifests prior to the onset of parkinsonism or within the first year of the disease, then the patient is classified as having DLB, whereas those parkinsonian patients who develop dementia beyond the first 12 months are regarded as having PDD [6, 7]. However, this temporal criterion oversimplifies the complex neuropathological landscape underlying these disorders. Both DLB and PDD involve Lewy body pathology, but they often coexist with other neurodegenerative changes such as Alzheimer’s disease pathology, contributing to significant heterogeneity. The strict reliance on timing risks obscuring this variability and may lead to misclassification, as patients with similar clinical presentations might have distinct underlying mechanisms. Moreover, the recently proposed biological frameworks have further complicated this scenario [8–10].

Both PDD and DLB manifest with a wide range of symptoms, encompassing neuropsychiatric, cognitive, motor, autonomic and sleep-wake disturbances. In particular, the neuropsychiatric symptoms in the LBDs represent a prominent driver in the loss of independent function, driving carer strain and an earlier transition to residential care [11–13]. Current evidence from multiple studies suggests that dementia with Lewy bodies (DLB) and Parkinson’s disease dementia (PDD) are closely related clinical syndromes, characterized by overlapping pathophysiology and similar trajectories of cognitive and motor decline. This supports their conceptualization as phenotypic variants of the same underlying Lewy body pathology. Longitudinal clinical data reveal no significant differences in the rates of change over six months in core clinical features such as global cognition (MMSE), motor parkinsonism (UPDRS-III), cognitive fluctuations, neuropsychiatric symptoms, and sleep disturbances between DLB and PDD. However, PDD patients exhibit more severe motor symptoms and require higher doses of dopaminergic medication at baseline [14]. Similarly, a large international cohort study reported comparable annual rates of cognitive decline and motor progression between DLB and PDD over a median follow-up of 2.5 years, with baseline motor severity being worse in PDD. Nonetheless, progression rates converged after adjusting for confounders, including age, sex, and dopaminergic therapy [15]. A motor performance study further corroborated this close relationship by analyzing DLB and PDD as Lewy body dementia (LBD), revealing broadly overlapping impairments in gait, balance, and hand dexterity relative to Parkinson’s disease without dementia and Alzheimer’s disease [16]. Despite the growing body of evidence indicating similarities between the two diseases, there is a paucity of studies examining the interconnections of their symptoms and their shared aetiology. A previous study demonstrated that symptoms clustered into distinct latent groups, suggesting a potential common pathological aetiology [17]. To further investigate these issues, we conducted a comprehensive phenotypic analysis of PDD and DLB to examine their symptom profiles and associations.

To address this gap, we conducted a detailed phenotypic analysis of PDD and DLB to investigate their symptom profiles and associations.

## Methods

Patients were recruited through the Parkinson's Disease Research Clinic in Sydney. The study protocol was approved by the Human Research Ethics Committee of Macquarie University (HREC #16842). All participants provided written informed consent prior to their involvement. Evaluations were conducted by qualified clinicians (SJGL, EM, CWG) using several scales, including the International Parkinson and Movement Disorder Society sponsored revision of the Unified Parkinson's Disease Rating Scale (MDS-UPDRS), the Scales for Outcomes in Parkinson’s Disease-Psychiatric Complications (SCOPA-PC), the Montreal Cognitive Assessment (MOCA), the Hospital Anxiety and Depression Scale (HADS), and the Epworth Sleepiness Scale (ESS). Motor subtypes were identified utilizing subsections of the MDS-UPDRS Section III, employing methodologies previously published in the literature [21].

Parkinson's Disease Dementia (PDD) was diagnosed according to the diagnostic criteria established by the Movement Disorders Society in 2007 [6]. All patients had a diagnosis of Parkinson’s disease for more than a year prior to developing cognitive impairment and had a MOCA score below 24 with deficits involving at least two cognitive domains. Patients with PDD were assessed either while they were on their medications (ON state) or off their medications (OFF state). All patients with Dementia with Lewy Bodies (DLB) were diagnosed according to the 4th DLB consensus criteria [7]. Dementia was defined as a progressive cognitive decline of sufficient magnitude to interfere with normal social or occupational functions, or with usual daily activities. Core features of DLB were assessed through clinical interviews, examination and polysomnography where possible. Polysomnography was available in a subset of participants to support the diagnosis of REM sleep behaviour disorder where clinically indicated. However, no additional biomarkers (e.g. cerebrospinal fluid analysis, PET imaging or α-synuclein seed amplification assays) were systematically collected in this cohort.

## Statistical methods

All statistical analyses were conducted using R version 4.4.1. Variables were treated as continuous or categorical according to their data type. Continuous variables are presented as mean (SD) and analysed using t-tests or Wilcoxon rank-sum tests, as appropriate. Categorical variables are presented as counts (percentages) and compared using chi-square or Fisher’s exact tests.

A correlation analysis was conducted to explore the relationship between key variables, including hallucinations (SCOPA PC Question 1), depression (HADS depression sub-score), apathy (MDS-UPDRS Question 1.5), motor severity (MDS-UPDRS Section III total), activities of daily living (ADL) score (MDS-UPDRS Section II), and the other phenotypic features (e.g. autonomic, neuropsychiatric features and fatigue). Standard bivariate correlations were calculated using Spearman's rank correlation coefficient (ρ) to assess monotonic relationships between all pairs of variables. No formal correction for multiple comparisons was applied in this analysis.

## Results

Data on 31 PDD and 30 DLB patients were available for analysis. As shown in Table 1, consistent with established epidemiological findings, patients were predominantly male in both groups (PDD Male 23[74%]; DLB Male 25[83%]; p=0.4). Both groups were matched for age at the time of assessment (PDD 75 ±9, DLB 75 ± 7; p>0.9). Age at diagnosis was significantly lower for PDD patients (60± 12 years) compared to DLB (73 ± 7; p<0.001) Data confirming the age at the onset of dementia was available for 15 PDD patients and did not differ from that of the age at which dementia was diagnosed in the 30 DLB patients (PDD 75 ± 6, DLB 74 ± 7; p=0.6). A higher proportion of DLB patients were assessed within 1 year of their dementia diagnosis compared to PDD (PDD 4/15(27%), DLB 19/30(63%); p=0.02).

**Table 1:** Demographics

Characteristic	PDD (n=31)	DLB (n=30)	p-value^*2*^	Overall N = 61^*1*^
Gender				
Female^1^	8 (26%)	5 (17%)	0.4	13 (21%)
Male^1^	23(74%)	25 (83%)		48 (79%)
Age at diagnosis^2^	60 (12)	73 (7)	<0.001	67 (12)
Years of disease^2^	15 (7)	1 (2)	<0.001	8 (9)
Age at assessment^2^	75 (9)	75 (7)	>0.9	75 (8)
Dementia diagnosis	PDD (n=15)	DLB (n=30)		
Age at dementia diagnosis^2^	75 (6)	74 (7)	0.6	74 (7)
Number of patients assessed within 1 year of dementia onset^1^	4 (27%)	19 (63%)	0.020	

^*1*^n (%)

^2^Mean (SD)

### Comparisons of cognitive and neuropsychiatric features

As shown in Table 2, the MoCA total score as a global measure of cognition demonstrated a non-significantly lower score in PDD patients compared to DLB (PDD 14.6 ± 6.2, DLB 17.3 ± 5.5; p=0.10). Psychotic features were significantly higher in PDD patients compared to DLB (visual hallucinations PDD 1.55±0.89, DLB 0.73; p <0.01, visual misperceptions PDD 1.52 ± 1, DLB 0.53 ± 0.82; p<0.001, delusions PDD 1.03 ± 1.02, DLB 0.53 ± 1.04; p=0.018). Overall, a higher proportion of PDD patients reported visual hallucinations (PDD 27/31 [87%], DLB 15/30[50%]; p<0.05) and delusions (PDD 19/31[61%], DLB 8/30[27% p<0.05). This was associated with a higher total SCOPA-PC score (PDD: 7.8 ± 3.7, DLB: 5.1 ± 4.1; p = 0.001). The PDD patients reported significantly more confusion and concentration difficulties as measured by SCOPA-PC question 5 (PDD 1.87 ± 0.72, DLB 1.50 ± 0.78; p = 0.047). There was no difference in HADS depression (PDD 7.3 ± 4.8, DLB 6.3 ± 4.2; p = 0.4) or anxiety scores (PDD 5.9 ± 3.7, DLB 5.9 ± 4.2; p=0.9) or the MDS UPDRS apathy score (PDD 1.5 ± 1.4, DLB 1.1 ± 0.92; p=0.2) between the groups. The PDD patients reported a significantly higher burden of autonomic dysfunction with more severe urinary dysfunction (PDD 2.74 ± 1.21, DLB 1.27 ± 1.17; p<0.001) and constipation (PDD 1.94 ± 1.12, DLB 0.97 ± 1.07; p<0.001), although there was no significant difference in orthostatic symptoms (PDD 1.19 ± 1.33, DLB 1.17 ± 1.02; p=0.7). Both fatigue (MDS-UPDRS Q1.13: PDD 2.3± 1.4, DLB 1.5 ± 1.0;p=0.006) and daytime somnolence (Epworth sleepiness scale total score PDD 15 ±6, DLB 10 ±5; p<0.001) were more severe in PDD in comparison to DLB.

**Table 2.** Phenotypic assessments

**Characteristic**	**PDD** N = 31^*1*^	**DLB**	**p-value**^*2*^	**Overall** N = 61^*1*^
MoCA Total^2^	14.6 (6.2)	17.3 (5.5)	0.10	15.9 (6.0)
Motor function				
MDS UPDRS Section III Total (Motor score)^2^	61 (17)	38 (16)	<0.001	50 (20)
Levodopa equivalent dose^2^	910 (544)	217 (396)	<0.001	569 (588)
Motor Phenotype			<0.001	
Indeterminate^1^	2(6.5%)	6(20%)		8(13%)
Postural Instability and Gait Difficulty phenotype (PIGD)^1^	29(94%)	17(57%)		46(75%)
Tremor Dominant Phenotype^1^	0(0%)	7(23%)		7
Autonomic function				
Urinary problems MDS-UPDRS Q1.10^2^	2.74 (1.21)	1.27 (1.17)	<0.001	2.02 (1.40)
Constipation MDS-UPDRS Q1.11^2^	1.94 (1.12)	0.97 (1.07)	0.001	1.46 (1.19)
Orthostatic symptoms - MDS-UPDRS Q1.12^2^	1.19 (1.33)	1.17 (1.02)	0.7	1.18 (1.18)
Fatigue, daytime somnolence and ability to complete activities of daily living				
Fatigue – MDS-UPDRS Q1.13^2^	2.3 (1.4)	1.5 (1.0)	0.006	1.90 (1.27)
Epworth Sleepiness Scale total score^2^	15 (6)	10 (5)	<0.001	12 (6)
MDS UPDRS Section II total (ADL activity)^2^	32 (9)	16 (8)	<0.001	24 (12)
SCOPA-PC questionnaire				
Hallucinations (Question 1)^2^	1.55 (0.89)	0.73 (0.87)	<0.001	1.15 (0.96)
Visual misperceptions (Question 2)^2^	1.52 (1.00)	0.53 (0.82)	<0.001	1.03 (1.03)
Delusions (Question 3)^2^	1.03 (1.02)	0.53 (1.04)	0.018	0.79 (1.05)
Question 4 – Altered dream phenomena^2^	1.32 (0.94)	1.00 (0.91)	0.2	1.16 (0.93)
Question 5 – Confusion^2^	1.87 (0.72)	1.50 (0.78)	0.047	1.69 (0.76)
Question 6 – Sexual preoccupation^2^	0.42 (0.72)	0.57 (0.90)	0.5	0.49 (0.81)
Question 7 – Impulse control behavior^2^	0.13 (0.43)	0.17 (0.59)	>0.9	0.15 (0.51)
Total score	7.8 (3.7)	5.1 (4.1)	0.001	6.5 (4.1)
Hospital anxiety and depression scale and apathy				
HADS Depression subscale^2^	7.3 (4.8)	6.3 (4.2)	0.4	6.8 (4.5)
HADS Anxiety subscale^2^	5.9 (3.7)	5.9 (4.2)	0.9	5.9 (3.9)
MDS-UPDRS Question 1.5 – Apathy^2^	1.5 (1.4)	1.1 (0.9)	0.2	1.31 (1.20)

^*1*^n (%)

^2^Mean (SD)

### Comparison of motor symptoms

Motor function was also significantly worse in PDD patients with a higher MDS-UPDRS section III score (PDD 61 ± 17; DLB 38 ± 16; p<0.001). The motor phenotypes exhibited significant differences between the groups, despite both predominantly displaying the postural instability and gait difficulty (PIGD) phenotype (PDD 29/31 [94%], DLB 17/30 [57%]; p<0.001). This was followed by the tremor-predominant phenotype (PDD 0/31 [0%], DLB 7/30 [23%]; p<0.001) and the indeterminate phenotype (PDD 2/31 [6.5%], DLB 6/30 [20%]; p<0.001).

### Comparison of therapies

Acetylcholinesterase inhibitor use was significantly higher in the DLB patients (PDD 8/31[26%], DLB 17/30[57%]; p=0.014), whilst the use of anti-psychotics (PDD 7/31[23%], DLB 4/30 [13%], p=0.3) and anti-depressants (PDD 9[29%], DLB [27%], p=0.8) was not significantly different between the groups. In DLB, only 17 patients (56%) were treated with dopaminergic therapy and consisted of a combination of monoamine oxidase B inhibitors and levodopa, whereas the PDD patients were treated with a range of therapies including levodopa (31/31 [100%], oral/topical dopamine agonists (8/31 [25.8%]), subcutaneous apomorphine (2/31 [6.4%]) and Levodopa-Carbidopa Intestinal Gel (1/31 [3.2%]). In addition, two patients with PDD had undergone deep brain stimulation.

### Associations with disease duration and dopaminergic therapy

It is important to highlight that there were no significant correlations identified between the length of the disease and any of the specified variables in either PDD or DLB. The levodopa equivalent daily dose (LEDD) did not correlate with any measures of psychosis or autonomic symptoms in either PDD or DLB. In the DLB group (n=30), a higher LEDD was positively correlated with motor severity (MDS‑UPDRS Section III ρ = 0.49, p <0.001), reduced activities of daily living (MDS‑UPDRS Section II ρ = 0.43, p <0.001), fatigue (MDS‑UPDRS Q1.13; ρ = 0.44, p <0.001) and apathy (MDS‑UPDRS Q1.5; ρ = 0.41, p <0.001. In the PDD group (n=31), higher LEDD was correlated with increased anxiety levels (HADS Anxiety score; ρ = 0.38, p <0.001) and increased compulsive behaviour (SCOPA‑PC Q7; ρ = 0.36, p,0.001).

### Associations with neuropsychiatric symptoms

Hallucinations (SCOPA-PC Question 1) showed similar correlations in both PDD and DLB, relating to increased visual misperceptions (PDD ρ=0.733, p<0.001; DLB ρ=0.62, p<0.01), delusions (SCOPA-PC Question 3: PDD ρ=0.57, p<0.001; DLB ρ=0.6, p<0.01), and altered dream phenomena (SCOPA-PC Question 4: PDD ρ=0.44, p<0.01; DLB ρ=0.39, p<0.01). Additionally, in DLB patients, hallucinations correlated with both poorer cognitive performance (MoCA total score ρ=−0.38, p<0.001), and depression (HADS depression subscale ρ=0.39, p<0.001), whilst in PDD there was a weak correlation with lower levels of fatigue (MDS UPDRS Question 1.13 ρ= −0.37, p<0.001).

Depression, as measured by the HADS depression subscale, demonstrated a correlation with anxiety (HADS Anxiety subscale: PDD ρ=0.54, p<0.001; DLB ρ=0.65, p<0.001) and delusions (SCOPA-PC Question 3: PDD ρ=0.47, p<0.001; DLB ρ=0.54, p<0.001). Furthermore, in DLB, depression exhibited a strong correlation with the broader hallucinatory phenotype, including hallucinations (SCOPA-PC Question 1: ρ=0.38, p<0.001), altered dream phenomena (SCOPA-PC Question 4: ρ=0.73, p<0.001), and confusion (SCOPA-PC Question 5: p<0.001). In PDD, depression was also correlated with (MDS-UPDRS Question 1.5: ρ=0.41, p<0.001) and sexual preoccupation (SCOPA-PC Question 7: ρ=0.35, p<0.001). In both PDD and DLB, apathy was correlated with fatigue (MDS-UPDRS Q1.13: PDD ρ=0.38, p<0.001; DLB ρ=0.48, p<0.001).

### Symptom correlations with function

Correlation analyses exploring the activities of daily living (ADLs) in PDD and DLB both demonstrated strong correlations with motor severity (PDD ρ= 0.65, p<0.001; DLB ρ = 0.52,p<0.001) and fatigue (PDD ρ = 0.58, p<0.001; DLB p = 0.45, p<0.001), as well as urinary symptoms (PDD ρ= 0.53,p<0.001;DLB ρ= 0.38,p<0.001). Whilst only showing a trend in PDD (ρ = 0.28; p = 0.06), impaired cognitive performance was significantly correlated with ADLs in DLB (PDD ρ = 0.47, p < 0.01). Additionally, DLB patients exhibited a significant correlation between ADLs and hallucinations (ρ = 0.39, p < 0.05), which was not observed in PDD. Conversely, PDD showed a significant correlation between ADLs and apathy (ρ = 0.42, p < 0.01), which was not evident in DLB.

## Discussion

In this detailed phenotyping study, we found that patients with PDD exhibited a more severe phenotype, characterized by significantly greater parkinsonism, psychosis, somnolence, fatigue, urinary disturbance and constipation compared to those with DLB. However, mood disturbance and orthostatic symptoms were similar across PDD and DLB. In both PDD and DLB, impaired ADLs were strongly correlated with motor impairment, fatigue and urinary symptoms. In PDD, apathy was also correlated with reduced ADLs, which was not observed in DLB. In contrast, ADLs in DLB were also impacted by hallucinations and impaired cognition, which was not evident in PDD.

Notably, the PDD and DLB groups in this study were evaluated at a similar age and although the PDD patients had a significantly longer disease duration, global cognitive scores did not differ significantly between the groups and the age at which dementia emerged was similar. Consequently, the phenotypic profiles presented here appear to represent two distinct groups of LBD patients with comparable cognitive abilities but clearly different patterns of disease progression, indicative of distinct pathophysiological progression.

Contrary to our findings, several studies have reported a greater prevalence of neuropsychiatric symptoms in DLB compared to PDD. Notably, some of these studies employed the Neuropsychiatric Inventory to assess neuropsychiatric symptoms, making direct comparisons with our own study challenging [18, 19, 22, 20]. Our research unit actively follows up patients with isolated RBD and screens for REM sleep without atonia using video polysomnography. These prodromal patients are followed up annually, allowing for the early detection of DLB, potentially before the onset of more florid psychotic phenomena. The observation that only 50% of the DLB patients in our cohort reported such symptoms (with 63% being assessed within one year of their DLB diagnosis) further supports this explanation. It is also possible that increased acetylcholinesterase inhibitor use in this population may have masked some of these symptoms [23].

In contrast, detecting cognitive decline in parkinson’s disease can be challenging given the difficulty of differentiating functional impairment from motor function versus that of cognitive decline. Furthermore patients with PD and their partners may therefore not report cognitive concerns promptly due to reasons such as care burden, pre-occupation with motor symptoms, and cognitive issues itself limiting communication [24]. In our experience caregivers are more inclined to address cognitive concerns when psychotic features emerge.

Both PDD and DLB demonstrated statistically significant correlations between misperceptions, delusions and altered dream phenomena, with DLB also showing a strong correlation with confusion. This observation further strengthens the notion of a broader hallucinatory phenotype that is associated with vivid dreams, REM Sleep behaviour disorder and attentional deficits [25–28]. These features have previously been linked to the cholinergic deficits observed in LBD [28, 29]. It is also interesting that in DLB a strong correlation was observed between depression and psychotic phenomena, which was not the case in PDD, despite having similar levels of treatment for mood disorder. This raises the possibility of a cholinergic impairment underlying depression in DLB, which has been suggested elsewhere [30].

The varying correlations between ADL scores (MDS-UPDRS Section II) and clinical symptoms in PDD and DLB may suggest underlying factors affecting daily functioning. Motor severity and fatigue showed robust correlations with ADL impairment in both groups, with larger effects in PDD. However, worse cognitive performance and hallucination severity demonstrated clearer associations with ADLs in DLB, with motor symptoms having a lesser impact. Conversely, in PDD, the strong association between motor symptoms and fatigue indicates that physical limitations may be the primary driver of reduced daily functioning, highlighting the need for tailored therapeutic approaches in each condition.

The observed differences between phenotypic features in PDD and DLB highlights the need for a nuanced distinction in our clinical appreciation of these disorders. Whilst both conditions share common pathological features, the varying patterns of neuropsychiatric symptoms suggest distinct underlying mechanisms or progression pathways. These phenotypic differences reflect variations in the spatial and temporal distribution of neuropathological changes within the brain [31]. Understanding these distinctions is crucial for improving diagnostic accuracy, tailoring treatment approaches and potentially developing targeted interventions that address the specific neuropsychiatric challenges faced by patients with each disorder.

This study has several important limitations that should be acknowledged. The limited sample size and the single-center design constrain the generalizability of the findings. The influence of levodopa dosage and the duration of the disease has not been comprehensively elucidated. Consequently, it remains uncertain whether the observed differences in severity between the two diseases are attributable to these variables, despite the absence of evidence supporting this in the correlation analysis. Additionally, our cross-sectional design limits our ability to establish temporal relationships between symptom clusters, lacking prospective longitudinal data that might help to clarify the evolution and course of symptoms across these two diseases. Future studies exploring the phenotype and underlying neurobiology should include additional biomarkers such as, cerebrospinal fluid analysis, PET imaging and α-synuclein seed amplification assays.

Despite these limitations, the current study supports the ongoing clinical distinctions between PDD and DLB. Future research should recognise this possibility and design larger, multi-centre prospective, longitudinal studies with detailed phenotyping combined with neuroimaging, neurophysiological, and fluid/tissue biomarkers. This work could potentially lead to improved diagnostic accuracy and targeted symptomatic and disease-modifying treatment strategies.

## Data Availability

Data are available from the corresponding author upon reasonable request.
